# Within‐person associations between psychological and contextual factors and lapse incidence in smokers attempting to quit: A systematic review and meta‐analysis of ecological momentary assessment studies

**DOI:** 10.1111/add.16173

**Published:** 2023-03-10

**Authors:** Olga Perski, Dominika Kwasnicka, Dimitra Kale, Verena Schneider, Dorothy Szinay, Gill ten Hoor, Bernard Yeboah‐Asiamah Asare, Peter Verboon, Daniel Powell, Felix Naughton, Jan Keller

**Affiliations:** ^1^ Department of Behavioural Science and Health University College London London UK; ^2^ Faculty of Psychology SWPS University of Social Sciences and Humanities Wroclaw Poland; ^3^ NHMRC CRE in Digital Technology to Transform Chronic Disease Outcomes, Melbourne School of Population and Global Health University of Melbourne Melbourne Australia; ^4^ Department of Work and Social Psychology, Faculty of Psychology and Neurosciences Maastricht University Maastricht the Netherlands; ^5^ Curtin School of Population Health Curtin University Perth Australia; ^6^ Health Psychology, Institute of Applied Health Sciences University of Aberdeen Aberdeen UK; ^7^ Faculty of Psychology Open University Heerlen the Netherlands; ^8^ Rowett Institute University of Aberdeen Aberdeen UK; ^9^ Behavioural and Implementation Science Research Group, School of Health Sciences University of East Anglia Norwich UK; ^10^ Department of Education and Psychology Freie Universität Berlin Berlin Germany

**Keywords:** Ambulatory assessment, ecological momentary assessment, meta‐analysis, smoking cessation, smoking lapse, systematic review

## Abstract

**Aims:**

When attempting to stop smoking, discrete smoking events (‘lapses’) are strongly associated with a return to regular smoking (‘relapse’). No study has yet pooled the psychological and contextual antecedents of lapse incidence, captured in ecological momentary assessment (EMA) studies. This systematic review and meta‐analysis aimed to synthesize within‐person psychological and contextual predictor–lapse associations in smokers attempting to quit.

**Methods:**

We searched Ovid MEDLINE, Embase, PsycINFO and Web of Science. A narrative synthesis and multi‐level, random‐effects meta‐analyses were conducted, focusing on studies of adult, non‐clinical populations attempting to stop smoking, with no restrictions on setting. Outcomes were the association between a psychological (e.g. stress, cravings) or contextual (e.g. cigarette availability) antecedent and smoking lapse incidence; definitions of ‘lapse’ and ‘relapse’; the theoretical underpinning of EMA study designs; and the proportion of studies with pre‐registered study protocols/analysis plans and open data.

**Results:**

We included 61 studies, with 19 studies contributing ≥ 1 effect size(s) to the meta‐analyses. We found positive relationships between lapse incidence and ‘environmental and social cues’ [*k* = 12, odds ratio (OR) = 4.53, 95% confidence interval (CI) = 2.02, 10.16, *P* = 0.001] and ‘cravings’ (*k* = 10, OR = 1.71, 95% CI = 1.34, 2.18, *P* < 0.001). ‘Negative feeling states’ was not significantly associated with lapse incidence (*k* = 16, OR = 1.10, 95% CI = 0.98, 1.24, *P* = 0.12). In the narrative synthesis, negative relationships with lapse incidence were found for ‘behavioural regulation’, ‘motivation not to smoke’ and ‘beliefs about capabilities’; positive relationships with lapse incidence were found for ‘positive feeling states’ and ‘positive outcome expectancies’. Although lapse definitions were comparable, relapse definitions varied widely across studies. Few studies explicitly drew upon psychological theory to inform EMA study designs. One of the included studies drew upon Open Science principles.

**Conclusions:**

In smokers attempting to stop, environmental and social cues and cravings appear to be key within‐person antecedents of smoking lapse incidence. Due to low study quality, the confidence in these estimates is reduced.

## INTRODUCTION

Cigarette smoking is one of the leading global causes of preventable ill‐health and death [1]. Supporting smokers to quit is a public health priority [2]. Smoking lapses (i.e. discrete smoking episodes during a quit attempt) are a key reason why smokers abandon their quit attempt and return to regular smoking [3, 4, 5]. Studies harnessing frequent, real‐time assessments in smokers’ daily lives (referred to as ecological momentary assessment; EMA) indicate that the risk of lapse incidence fluctuates over time within individuals and is influenced by different psychological and contextual factors [6, 7, 8, 9, 10, 11, 12]. A multitude of theoretical frameworks and models have attempted to explain and predict when and why lapses will occur. According to the Negative Reinforcement Model of Addiction (which exists in several formulations), avoidance of negative affect and discomfort through smoking is the key driver of lapses [13]. According to the Relapse Prevention Theory (sometimes referred to as the Cognitive–Behavioural Relapse Model), lapses are driven by encountering high‐risk situations (e.g. specific emotional or physiological states and environmental cues), which ‘force’ the person to amount a coping response to try to avoid a lapse, with the specific response mounted being more or less successful (depending on whether or not a lapse is avoided) [14, 15]. This is followed by the appraisal of the lapse event, which can be more or less damaging for the person’s self‐efficacy (e.g. due to self‐blame), often setting the person on a course towards full relapse. Other, slightly differently formulated theoretical frameworks include those focused on self‐regulation (e.g. the Strength Model of Self‐Regulation), which posit that the self‐control (or other coping resources) required to resist temptations to smoke depletes over time (also referred to as ‘cessation fatigue’), thus making the individual increasingly vulnerable to lapsing [16]. Data from EMA studies show that lapses tend to occur rapidly—i.e. within 11 minutes [17]—due to acute bouts of intense cravings following exposure to psychological or contextual cues that have become associated with smoking through a process of conditioning (e.g. a lit up cigarette, stress or negative affect) [6, 7, 8, 9, 10, 11, 12]. Typically, however, multiple conditions must align for lapses to occur—e.g. stress‐ or affect‐induced cravings at a time when cigarettes are easily available—and the specific psychological and contextual cues that increase lapse risk differ between individuals [6, 7, 8, 9, 10, 11, 12], highlighting the need for tailored, real‐time lapse prevention support.

Available systematic reviews have synthesized evidence on motives for substance use in EMA studies [18] and compliance with EMA protocols in studies focused on substance use (including cigarette smoking) [19]. However, we currently lack a comprehensive review and synthesis of EMA studies that examine within‐person associations between psychological (e.g. negative affect, cravings or positive affect) or contextual cues (e.g. the presence of other smokers or cigarette availability) and smoking lapse incidence. To provide a valid assessment of factors which may most compromise a quit attempt by raising lapse risk, it is important to focus on studies investigating within‐person associations among smokers attempting to quit (as opposed to when smoking *ad libitum*). Such findings would provide a useful resource for researchers and practitioners interested in the development and evaluation of tailored smoking cessation interventions, particularly ‘just‐in‐time adaptive interventions’ (JITAIs), which aim to provide the right type of support to smokers at the right time [20, 21, 22]. In this review we focus primarily on lapse incidence (rather than relapse), given the vital role of lapses in setting the individual on a course towards a return to regular smoking (although see the paragraph below where we focus specifically on relapse).

We also have limited knowledge regarding (i) how EMA researchers have defined ‘lapse’ and ‘relapse’ in EMA studies, (ii) the theoretical underpinning of EMA study designs and (iii) the proportion of published EMA studies with pre‐registered study protocols and open data. First, the Society for Research on Nicotine and Tobacco (SRNT) treatment research network has recently published recommendations for abstinence definitions in clinical trials of smoking cessation interventions. They note that abstinence definitions ‘vary in how they address the realities of the quitting process’, including whether definitions allow for a few lapses prior to achieving long‐term abstinence [23]. However, this raises the question of how to distinguish lapses from full‐blown relapse. Such a distinction is necessary to develop evidence‐informed, real‐time lapse prevention support (e.g. JITAIs). The SRNT treatment network suggests that relapse is defined as follows: ‘a return to regular smoking following a period of abstinence (i.e. seven consecutive days of smoking)’ [23]. As EMA studies allow much closer, real‐time monitoring of lapse patterns over time than traditional pre–post study designs, it is important to examine what lapse and relapse definitions have been used in published EMA studies, and what implications this has for the future development of real‐time lapse prevention support.

Second, EMA studies—due to their ability to capture the dynamics of smoking behaviour in context—are uniquely placed to test and potentially refine available psychological theories of addiction and behaviour change to more effectively account for the observed dynamic nature of addictive behaviours. It has been argued that health behaviour theories must apply to individuals [24], but most studies that aim to test or develop health psychology theory have traditionally focused on why people differ from one another (i.e. between‐ rather than within‐person differences) [25, 26]. It is currently unclear whether published EMA studies have leveraged the opportunity to test or update available theories of addiction or behaviour change, including, but not limited to, the theoretical frameworks and models mentioned above (e.g. the Negative Reinforcement Model of Addiction, the Relapse Prevention Theory and the Strength Model of Self‐Regulation). For example, in a typical EMA study, participants are prompted several times per day to respond to a brief survey with questions about how they are feeling (e.g. sad or stressed), what they are doing (e.g. being around other smokers), what is in their immediate context (e.g. whether cigarettes are easily available), who they are with (e.g. alone or with a friend) and whether they have smoked. Next, the relationship of these variables at *t*_1_ with lapse incidence reported at *t*_2_ is modelled—typically using a multi‐level model, which can account for the nested measurements within the same individuals. Hence, EMA studies provide a unique opportunity to explicitly test hypotheses stemming from available theoretical frameworks (e.g. are lapses primarily driven by negative affect, rather than cravings triggered by environmental cues, as posited by the Negative Reinforcement Model of Addiction?).

Third, as EMA researchers face many study design and analytical decisions, such as selecting the EMA prompting frequency, the type of statistical model to use and what parameters to include (e.g. random intercepts and slopes to account for inter‐individual differences), pre‐registration of study protocols and analytical plans via public repositories such as the Open Science Framework (www.osf.io) is important for replicability and reproducibility [27, 28]. In addition, given the rapidly growing number of EMA studies published each year [29] and the relatively high cost of EMA designs (including participant burden), it would be useful for researchers and intervention designers to be able to re‐use data from previous studies. However, the extent to which available EMA studies align with principles of the Open Science movement is currently unknown.

The aims of this systematic review and meta‐analysis were therefore:

1. To synthesize within‐person associations between psychological (e.g. craving) or contextual factors (e.g. the presence of other smokers) and smoking lapse incidence in healthy (i.e. non‐clinical), adult smokers attempting to quit;
2. To summarize how EMA researchers have defined ‘lapse’ and ‘relapse’;
3. To summarize the theoretical underpinning of EMA study designs; and
4. To summarize the proportion of studies with pre‐registered study protocols/analysis plans and open data.

## METHODS

### Study design

This was a systematic review and meta‐analysis, which formed part of a larger, systematic review of EMA studies of five key public health behaviours (i.e. physical activity and sedentary behaviour, dietary behaviour, alcohol consumption, tobacco smoking and sexual health) [29, 30]. The Preferred Reporting Items for Systematic Reviews and Meta‐Analyses (PRISMA) checklist and the American Psychological Association’s Meta‐Analysis Reporting Standards [31] were used in the design and reporting of this systematic review [32], with the review protocol being pre‐registered on the Open Science Framework (https://osf.io/49uqf/).

### Inclusion criteria

We included EMA studies that recruited as participants tobacco smokers (e.g. cigarettes, cigar and pipe) aged 18+ years who were undergoing a quit attempt. No restrictions on geographical location or publication date were set. Studies needed to include multiple (i.e. two or more) EMAs collected at a regular frequency up to 1 week apart of at least one EMA‐measured psychological or contextual predictor and smoking lapse incidence, and to have reported one or more within‐person predictor–lapse association(s). We note that some predictor–lapse associations may involve same‐time (rather than lagged) EMAs of psychological/contextual variables and lapse incidence. For these studies, the term ‘correlate’ rather than ‘antecedent’ or ‘predictor’ may be more appropriate. However, for ease of reporting, we henceforth refer to both as ‘antecedents’ or ‘predictors’. Observational or experimental studies harnessing self‐reported, smartphone‐ or external sensor‐assessed, physiological (e.g. heart rate variability to capture stress) or meteorological measures (e.g. weather data) of psychological and/or contextual predictors and smoking lapse were included.

### Exclusion criteria

Studies where participants were recruited based on being diagnosed with a physical or mental health condition such as cancer, cardiovascular disease, depression, binge eating disorder or substance use disorder were excluded as per the larger systematic review [30]. As we anticipated many relevant studies in the larger review, a decision was made to focus only on adult, non‐clinical populations to limit the scope. In addition, studies were excluded if they addressed *adlibitum* tobacco smoking but did not focus on lapse incidence (binary) in smokers undergoing a quit attempt.

### Search methods for the identification of studies

#### Electronic searches

We searched Ovid MEDLINE, Embase, PsycINFO and Web of Science (see the Supporting information for the full search strategy). Terms were searched for in titles and abstracts as free text terms or as index terms (e.g. Medical Subject Headings), as appropriate. We combined two groups of terms: the first group included terms relevant to EMAs and within‐person study designs, the second group included terms relevant to smoking [29]. Electronic and hand‐searches were conducted in January 2020 and updated in February 2021. As a result of the peer review process the search terms were expanded, and the electronic searches were updated in November 2022 (see the Supporting information for the updated search strategy). We restricted the search to human studies available in English that were published in peer‐reviewed journals.

#### Searching for other sources

Reference lists of available systematic reviews of EMA studies were hand‐searched (in January 2020 and February 2021) and expertise within the review team was used to identify additional articles of interest.

### Data collection and analysis

#### Selection of studies

Identified records were merged using Covidence [33] and duplicate records were removed. Two reviewers (O.P. and D.Kw.) independently screened titles and abstracts (‘yes’, ‘maybe’ and ‘no’) against the inclusion criteria. As part of the updated search in November 2022, titles and abstracts were screened by O.P. with 10% independently screened by other reviewers from the larger review team (D.K., J.K. and G.t.H.). Full texts were independently screened by two reviewers from the larger review team (yes and no); discrepancies were resolved by three reviewers (O.P., J.K. and D.Kw.) and inclusion was further discussed with other team members if needed. As part of the updated search in November 2022, full texts were screened by O.P. with 10% independently screened by another reviewer from the larger review team (D.K.). We did not calculate inter‐rater reliability. In line with the PRISMA checklist, a primary reason for exclusion for each study was recorded at the full text stage. Exclusion criteria were hierarchically ordered and included: a full text could not be obtained; study protocol; study not published in English; conference abstract; duplicate; wrong study design (i.e. not an EMA study); participants recruited based on a physical or mental health condition; participants younger than 18 years; study did not focus on smoking lapse incidence; and study did not report a within‐person psychological/contextual predictor–lapse association.

#### Data extraction and management

A data extraction form was developed in Microsoft Excel by three reviewers (O.P., D.Kw. and J.K.) in collaboration with the larger review team to extract information on participant characteristics; smoking characteristics; psychological/contextual predictors assessed; EMA delivery mode; EMA prompting method; EMA sampling frequency; authors’ definitions of ‘lapse’ and ‘relapse’; authors’ explicit (rather than inferred) descriptions of the theory/theories underpinning the EMA study design (here, we took an inclusive approach to the definition of theory: ‘A theory presents a systematic way of understanding events or situations. It is a set of concepts, definitions, and propositions that explain or predict these events or situations by illustrating the relationships between variables’ [34]); whether psychological theory was used to inform the psychological or contextual variables assessed (‘yes’ vs. ‘no’), the EMA sampling frequency (‘yes’ vs. ‘no’) or the study duration (‘yes’ vs. ‘no’); whether the study protocol had been pre‐registered (‘yes’ vs. ‘no’); whether the data underpinning the analyses had been made openly available via a public repository (‘yes’ vs. ‘no’); and details regarding the statistical analysis (e.g. within‐person model coefficients and standard errors, the type of statistical model used and whether the modelled predictor–lapse association pertained to a same‐time or lagged relationship; see the Supporting information). If multiple statistical models were reported, the model with the greatest number of parameters was selected and the respective covariate types and names were extracted. Within‐person effect sizes and standard errors were extracted directly from the results sections of the included studies (e.g. tables with model coefficients or in‐text model summaries).

Data were extracted by one reviewer (O.P.), with 20% of studies double‐checked by a second reviewer (G.t.H.) for accuracy and completeness. In addition, 100% of the within‐person model coefficients and standard errors were double‐checked by a third reviewer (D.S.) for accuracy and completeness. Discrepancies were resolved through discussion, consulting the senior author (J.K.) if required. As part of the updated search in November 2022, data were extracted by one reviewer (O.P.), with 10% double‐checked by a second reviewer (J.K.) for accuracy and completeness. In addition, the same reviewer double‐checked 100% of the within‐person model coefficients and standard errors for accuracy and completeness.

#### Quality appraisal

As no fit‐for‐purpose quality appraisal tool was identified prior to conducting the larger EMA review, we amended an available checklist for the reporting of EMA studies [35] to include the following four criteria: rationale for using the EMA design (quality 1); whether an a priori power analysis had been conducted (quality 2); adherence to the EMAs (quality 3); and treatment of missingness (quality 4). We applied a standardized classification system based on the Effective Public Health Practice Project quality assessment tool [36] by rating the quality of each EMA study according to the abovementioned criteria as ‘weak’, ‘moderate’ or ‘strong’ [30]. For the studies included in the present review, the four quality indicators were coded by one reviewer (O.P.), with 20% double‐checked for accuracy and completeness by a second reviewer (G.t.H.). Discrepancies were resolved through discussion and by consulting a third reviewer (D.P.) if required. As part of the updated search in November 2022, the quality indicators were coded by one reviewer (D.K. or V.S.), with 10% double‐checked for accuracy and completeness by a second reviewer (G.t.H.).

### Data synthesis

#### Data pre‐processing

The psychological and contextual variables extracted were coded against the following higher‐order categories [29], developed by three reviewers (O.P., D.Kw. and J.K.) based on the Theoretical Domains Framework [37]: ‘feeling states—unspecified’, ‘positive feeling states’, ‘negative feeling states’, ‘momentary trait manifestations and physical states’, ‘motivation and goals’, ‘beliefs about capabilities’, ‘beliefs about consequences’, ‘behavioural regulation’, ‘memory, attention and decision processes’, ‘social influences’, ‘environmental context and physical/environmental resources’ and ‘nature of the behaviour’. The psychological and contextual variables were coded by one reviewer (O.P.) and double‐checked by two reviewers (D.Kw. and J.K.). Discrepancies were resolved through discussion among three reviewers (O.P., D.Kw. and J.K.). Prior to conducting the meta‐analyses, finer groupings of the psychological and contextual variables were generated (see the Supporting information). For example, ‘urges’ and ‘motivation to stop’ had initially been coded under the higher‐order construct ‘motivation and goals’ but were separated prior to the meta‐analysis, as they capture different motivational processes (i.e. motivation to smoke and motivation not to smoke, respectively). As part of the updated search in November 2022, psychological and contextual variables were coded by one reviewer (O.P.), with 10% double‐checked by a second reviewer (J.K.).

Inductive thematic analysis was used to organize the extracted data pertaining to study authors’ definitions of ‘lapse’ and ‘relapse’ and descriptions of the theory underpinning the EMA study design into higher‐order categories [38]. Definitions and descriptions were coded by one reviewer (O.P.), with 20% double‐checked by a second reviewer (F.N. and J.K.). Discrepancies were resolved through discussion. Next, similar codes were grouped together into higher‐order thematic categories by one reviewer (O.P.) and double‐checked by a second reviewer (F.N. and J.K.). As part of the updated search in November 2022, definitions and descriptions were coded by one reviewer (O.P.), with 10% double‐checked by a second reviewer (J.K.).

The data and R code underpinning the analyses are available via GitHub (https://github.com/OlgaPerski/EMA_smoking_lapse_review).

#### Identifying duplicate samples

Although we did not systematically extract information on overlapping samples among included studies at the time of data extraction for the initial searches (January 2020 and February 2021), we returned to the data set to identify such samples using the following approach: (i) two reviewers (D.P. and F.N.) flagged studies with identical sample sizes and identical sample mean ages; and (ii) checked the author list for overlaps in co‐authorship. Where (i) and (ii) were satisfied, studies were coded as having an overlapping sample and the earliest study was included. Where an overlap in co‐authorship was not identified, the article full texts were further checked. Next, the ‘general comments’ column in the data extraction sheet (used by the reviewers to highlight any queries) was screened for any mention of overlapping samples, and where this was the case this was confirmed by checking if the samples in the articles were the same or a subsample of each other. Finally, where the first approach brought up sample sizes and mean ages that were very close but not identical, the articles were further screened to check for overlapping samples, keeping the earliest record of a study using each sample. As part of the updated search in November 2022, articles were screened for overlapping samples during the full text stage (O.P.) and excluded prior to the data extraction.

#### Narrative synthesis

A narrative synthesis was conducted to summarize the characteristics of all the included studies and the within‐person predictor–lapse associations which could not be included in the meta‐analyses. Results pertaining to the predictor–lapse associations were grouped by the type of predictor and presented if at least two effect sizes pertaining to the same predictor variable were available.

#### Meta‐analysis

We had pre‐specified in the review protocol that if a sufficient number of studies (i.e. ≥ 5) with similar within‐person psychological or contextual predictor variables were identified—based on the higher‐order construct categories specified above—multi‐level, random‐effects meta‐analyses of within‐person associations would be conducted in the R software environment with the *metafor* and *robumeta* packages [39, 40] due to the potential nesting of effect sizes within studies. However, following statistical review by P.V., we instead considered ≥ 10 studies with similar within‐person predictor variables sufficient for meta‐analysis to ensure adequate power. In all models, the unit of analysis was lapse incidence since the previous daily or hourly EMA report. Following the review process, in the event of a simpler model (e.g. a two‐ rather than three‐level model) being able to provide an equally good explanation of the data, we opted for the simplest possible model.

To explore heterogeneity, the *I* ^2^ statistic was calculated, and where at least some heterogeneity was observed (i.e. *I* ^2^ > 1%), moderator analyses were performed through meta‐regression. We did not have any pre‐specified hypotheses regarding the potential moderators; all variables were entered simultaneously. The selection of moderator variables to include was guided by the observed variability across studies. We included the following moderator variables: baseline age, sex and ethnicity; baseline cigarettes per day; study design (i.e. observational vs. interventional); study duration in days; incentive schedule (i.e. flat payment vs. multiple vs. other vs. payment per EMA vs. not reported); and whether a random slope had been specified (no vs. yes vs. not reported). For variables with missing data (i.e. baseline age, sex, ethnicity and cigarettes per day), median imputation was used.

Risk of bias due to missing results, potentially reflecting reporting biases, was explored with funnel plots and Egger’s test by entering the sampling variance as a moderator variable in the multi‐level, random‐effects meta‐analyses [41]. Sensitivity analyses with robust variance estimation were conducted, which accounted for the non‐independence of effect sizes when multiple effect sizes from single studies were pooled, without requiring access to information regarding within‐study correlations [40]. In these analyses, rho was set to 0.8 (the default value in the R package), as we did not have any pre‐specified hypotheses regarding how strongly correlated the effect sizes would be. However, unplanned sensitivity analyses (following the review process) in which we varied rho systematically (0.2, 0.4, 0.6 and 0.8) yielded identical results, probably due to the small number of effect sizes nested within the same study in our analyses (see the Results section). In addition, where relevant, following inspection of the forest plots, leave‐one‐out sensitivity analyses (unplanned) were conducted to examine the influence of one or more large effect sizes on the overall pooled estimate [42].

## RESULTS

**FIGURE 1:**
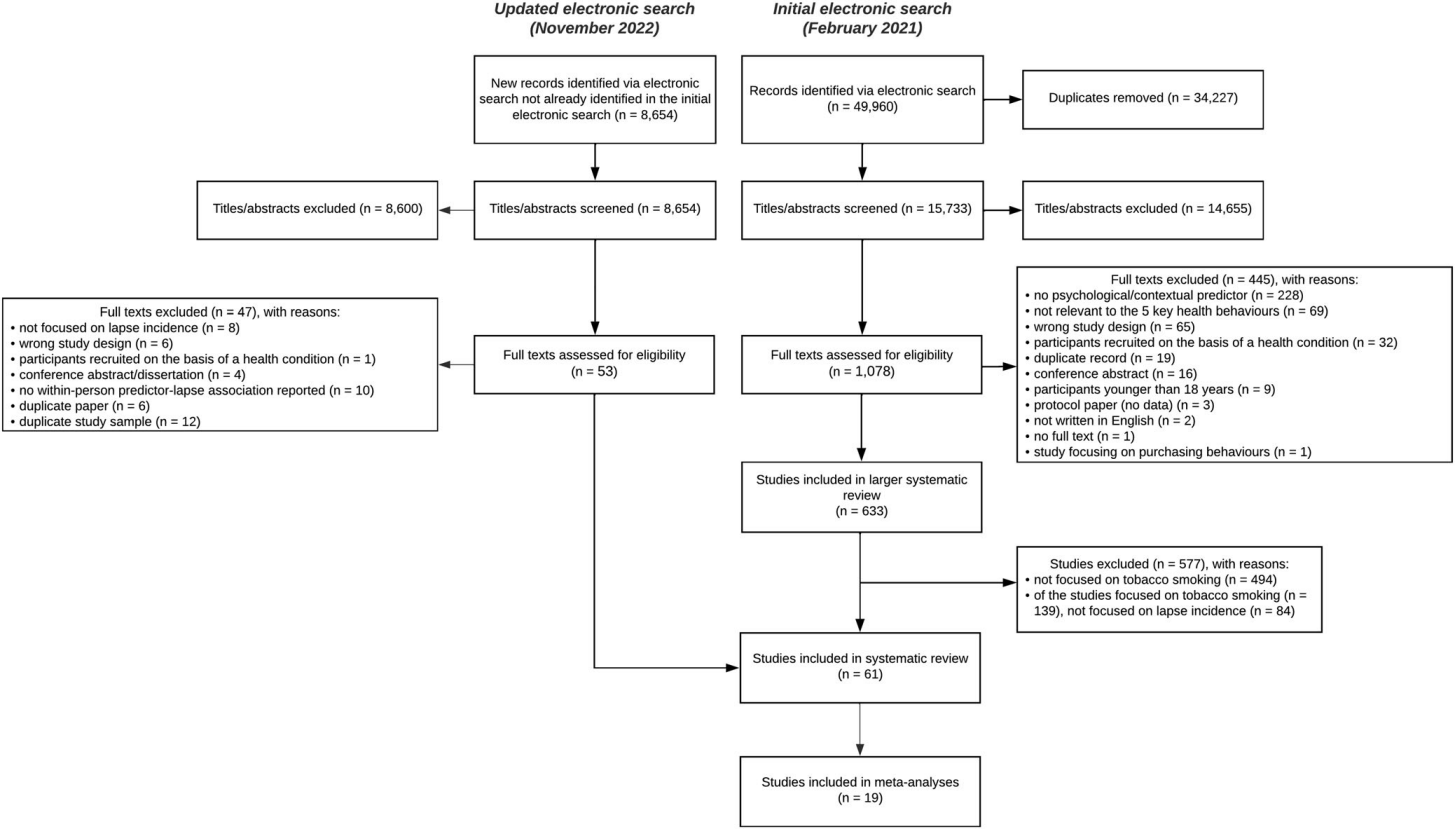
Preferred Reporting Items for Systematic Reviews and Meta‐Analyses (PRISMA) flow diagram of included studies.

After removing duplicates, 15 733 records were identified as part of the larger review, with 1078 studies screened at the full text stage. Of the 633 studies included in the larger review, 139 (139 of 633; 22%) focused on tobacco smoking. Of these, 55 (55 of 139; 39.5%) studies reported among 56 articles focused on lapse incidence and were included in the present review. Following the updated search in November 2022 another six studies were added, resulting in a total of 61 included studies reported among 62 articles. Nineteen studies (19 of 61; 31%) reported among 20 articles provided effect sizes that could be included in the meta‐analyses (see Figure 1).

### Study characteristics

Most studies were conducted in the United States (56 of 61; 93.3%) and primarily received funding from research/government organizations (50 of 61; 82.0%; see Table 1). Studies reported a median (Q1 and Q3) sample size of 198.0 (92.0, 325.0) participants who were aged a median of 42.0 (39.2, 44.2) years. Studies included a median of 55.6% (50.1, 58.0) women, with 84.0% (53.5, 89.2) of participants identifying as white ethnicity and 43.0% (37.0, 74.7) with a university degree. At baseline, participants smoked a median (Q1 and Q3) of 21.4 (18.6, 24.5) cigarettes per day and had made 3.9 (3.2, 4.8) quit attempts. Most studies recruited participants from the general population (56 of 61; 91.8%). Most studies used interventional rather than observational designs (41 of 61; 67.2%). A minority of studies did not report the use of incentives for participation or data completion (25 of 61; 41.0%). The remaining studies reported the use of some form of incentive, including, but not limited to, flat payment based on study completion (10 of 61; 16.4%), multiple incentives (nine of 61; 14.8%) or payment per EMA (six of 61; 9.8%; see Table 1).

**TABLE 1:** Study characteristics.

	*n* = 61
Country	
United States	56 (93.3%)
Netherlands	2 (3.3%)
Switzerland	2 (3.3%)
Not reported	1 (1.6%)
Funding source	
Research/government funding^a^	50 (82.0%)
Society funding^a^	4 (6.6%)
Charity funding^a^	9 (14.8%)
University/health institution funding^a^	3 (4.9%)
Industry funding^a^	4 (6.6%)
No funding	9 (14.8%)
Study design	
Observational	20 (32.8%)
Interventional	41 (67.2%)
Intervention level	
Between‐person (group level)	39 (63.9%)
Within‐person (person level)	1 (1.6%)
Mixed	1 (1.6%)
Not applicable	20 (32.8%)
Population type	
General population	56 (91.8%)
Heterosexual couples	1 (1.6%)
Other	4 (6.6%)
Sample size	
Median	198.0
Q1, Q3	92.0, 325.0
Age, mean	
Median	42.0
Q1, Q3	39.2, 44.2
Not reported	2
% Female	
Median	55.6
Q1, Q3	50.1, 58.0
Not reported	5
% White ethnicity	
Median	84.0
Q1, Q3	53.5, 89.2
Not reported	13
% University education	
Median	43.0
Q1, Q3	37.0, 74.7
Not reported	39
Cigarettes per day, mean	
Median	21.4
Q1, Q3	18.6, 24.5
Not reported	16
Number of quit attempts, mean	
Median	3.9
Q1, Q3	3.2, 4.8
Not reported	45
Smoking cessation support	
Behavioural support only	10 (16.4%)
Pharmacological support only	11 (18.0%)
Both behavioural and pharmacological support	13 (21.3%)
No support/not specified	27 (44.3%)
Incentive schedule	
Flat payment based on study completion	10 (16.4%)
Multiple	9 (14.8%)
Other	11 (18.0%)
Payment per EMA	6 (9.8%)
No/not reported	25 (41.0%)

Abbreviation: EMA, ecological momentary assessment.

^a^ Not mutually exclusive.

### EMA characteristics

The median (Q1 and Q3) study duration was 28.0 (14.0, 35.0) days (see Table 2). None of the included studies used a burst design. In most studies, none of the participants used their own device (i.e. all participants were provided with a study‐specific EMA device) (50 of 61; 82.0%). EMAs were primarily delivered via hand‐held devices (39 of 61; 63.9%). The most commonly used EMA sampling method was ‘multiple’ (i.e. a combination of signal and event contingent sampling; 40 of 61; 65.6%). The most commonly used EMA sampling frequency was multiple times per day (55 of 61; 91.8%). The median (Q1 and Q3) percentage of EMA adherence was 77.4% (75.1%, 85.5%). The majority of studies reported using an adherence cut‐off for inclusion of participants in the data analyses (32 of 61; 52.5%).

**TABLE 2:** EMA characteristics.

	*n* = 61
Study duration (days)	
Median	28.0
Q1, Q3	14.0, 35.0
Burst design	
No	61 (100.0%)
% Own device	
All participants	5 (8.2%)
Some participants	1 (1.6%)
None of the participants	50 (82.0%)
Not applicable	2 (3.3%)
Not reported	3 (4.9%)
% EMA delivery mode	
Hand‐held device	39 (63.9%)
Mobile phone—app	8 (13.1%)
Mobile phone—multiple/other	4 (6.6%)
Mobile phone—SMS	2 (3.3%)
Multiple	2 (3.3%)
Other	1 (1.6%)
Pen‐and‐paper	2 (3.3%)
Not reported	3 (4.9%)
% Adherence	
Median	77.4
Q1, Q3	75.1, 85.5
Not reported	27
Adherence cut‐off	
No	28 (45.9%)
Yes	32 (52.5%)
Not reported	1 (1.6%)
% EMA sampling frequency	
Daily	4 (6.6%)
Multiple times per day	56 (91.8%)
Hourly	1 (1.6%)
% EMA sampling method	
Event contingent	1 (1.6%)
Fixed (e.g. every evening)	4 (6.6%)
Multiple	40 (65.6%)
Signal contingent—random timing	15 (24.6%)

Abbreviations: EMA, ecological momentary assessment; SMS, short message service.

### Study quality

Studies generally received a ‘strong’ rating for quality 1 (i.e. rationale provided for the EMA design; see Table 3), a ‘weak’ rating for quality 2 (i.e. whether an a priori power analysis had been conducted) and a ‘weak’ rating for quality 4 (i.e. treatment of EMA missingness). For quality 3 (i.e. adherence to the EMAs), ratings were more evenly split across ‘weak’, ‘moderate’ and ‘strong’.

**TABLE 3:** Quality of included studies.

	*n* = 61
Quality 1—rationale for the EMA design	
Weak	1 (1.6%)
Moderate	3 (4.9%)
Strong	57 (93.4%)
Quality 2—whether an a priori power analysis had been conducted	
Weak	59 (96.7%)
Moderate	0 (0.0%)
Strong	2 (3.3%)
Quality 3—adherence to the EMAs	
Weak	25 (41.0%)
Moderate	18 (29.5%)
Strong	12 (19.7%)
Not reported	6 (9.8%)
Quality 4—treatment of missingness	
Weak	54 (88.5%)
Moderate	7 (11.5%)
Strong	0 (0.0%)

Abbreviation: EMA, ecological momentary assessment.

### Definitions of ‘lapse’ and ‘relapse’

Forty‐eight of the included studies (48 of 61; 78.7%) provided a definition of a ‘lapse’. Of these, definitions were coded under the following higher‐order categories: ‘any smoking after the quit date’ (35 of 48; 72.9%), ‘any smoking since the last report’ (six of 48; 12.5%), ‘smoking at least one cigarette since the last report’ (five of 48; 10.4%) and ‘any smoking over a defined time‐frame’ (two of 48; 4.2%). See the Supporting information for a list of the definitions provided by the study authors.

Thirty‐three of the included studies (33 of 61; 54.1%) provided a definition of ‘relapse’. Of these, definitions were coded under the following higher‐order categories: ‘threshold’ (16 of 33; 48.5%; e.g. ‘five or more cigarettes on 3 consecutive days’ and ‘7 consecutive days of smoking’), ‘undefined regular smoking’ (eight of 33; 24.2%; e.g. ‘a return to regular smoking’ and ‘falling back to smoking’), ‘any smoking after the quit date’ (five of 33; 15.2%; e.g. ‘at least one cigarette puff after the quit date’) or ‘stopped trying’ (four of 33; 12.1%; e.g. ‘no longer trying to refrain from use’). See the Supporting information for a list of definitions provided by the study authors.

### Theoretical underpinning of EMA study designs

Thirty‐four of the included studies (34 of 61; 55.7%) mentioned the use of at least one psychological theory, which were coded under the following higher‐order categories: Relapse Prevention Theory (13 of 34; 38.2%), the Negative Reinforcement Model of Addiction (seven of 34; 20.6%), the Strength Model of Self‐Regulation (three of 34; 8.8%), the Model of Absent‐Minded Lapses (two of 34; 5.9%), Reversal Theory (two of 34; 5.9%), Social Learning Theory (two of 34; 5.9%), the Broaden‐and‐Build Theory of Positive Emotion (two of 34; 5.9%), Attentional Bias Theory (one of 34; 2.9%), the Episodic Model of Relapse (one of 34; 2.9%) and Expectancy Violation Theory (one of 34; 2.9%). See the Supporting information for a list of theories mentioned by the study authors. In the studies where theory was mentioned, all were judged to have drawn upon the theory to inform the psychological or contextual variables assessed (34 of 34; 100%), with one study drawing on theory to inform the EMA frequency (one of 34; 2.9%) and none of the studies drawing on theory to inform the study duration (none of 34; 0%).

### Pre‐registration of study protocols/analysis plans and open data

One of the included studies (one of 61; 1.6%) reported pre‐registering their study protocol on a publicly available platform (e.g. the Open Science Framework). One of the included studies (one of 61; 1.6%) had made the study data openly available via a public repository.

### Psychological and contextual predictors of momentary smoking lapse incidence

**FIGURE 2:**
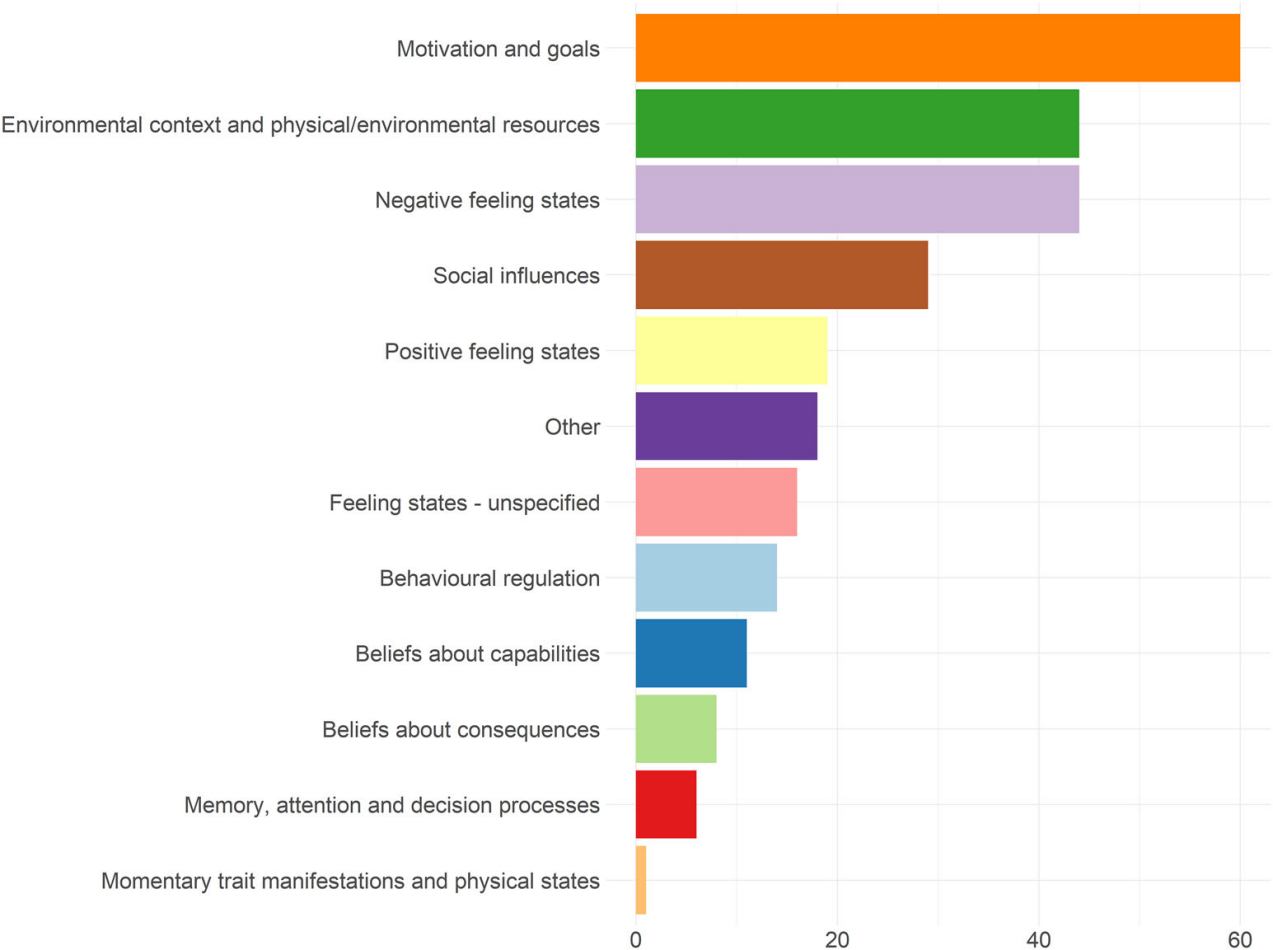
Frequency plot of the psychological and contextual predictors of lapse incidence.

The included studies examined a median (Q1 and Q3) of four (two, seven) psychological or contextual lapse predictors (range = 1–12; total across the included studies = 270). The most frequently assessed constructs were ‘motivation and goals’ (60 of 270; 22.2%), ‘negative feeling states’ (44 of 270; 16.3%) and ‘environmental context and physical/environmental resources’ (44 of 270; 16.3%) (see Figure 2). Of the psychological and contextual predictors assessed, a minority (42 of 270; 15%) were reported to be measured with a single item (versus multiple items versus not reported). A minority (37 of 270; 13.7%) were reported to have been measured with items for which there was a precedent (i.e. the items having previously been used in an EMA study versus items being developed specifically for the study versus the item origin not being reported).

### Summary of statistical models and model parameters

Twenty‐one studies jointly reported 63 effect sizes pertaining to within‐person predictor–lapse associations, with nineteen studies contributing ≥ 1 effect size(s) to the meta‐analyses. Momentary lapse incidence was assessed multiple times per day via self‐report without the use of carbon monoxide monitors or passive sensors (63 of 63; 100%). Most effect sizes were estimated with hierarchical or multi‐level regression models (51 of 63; 81.0%), followed by multi‐level structural equation models (12 of 63; 19.0%). Most effect sizes were modelled as part of multi‐ rather than univariable models (61 of 63; 96.8%) and the method for managing missing data was most commonly coded as ‘not reported’ (40 of 63; 63.5%). Where the method for managing missing data had been specified (23 of 63; 36.5%), maximum likelihood techniques were used (23 of 23; 100%). Predictor–lapse associations were primarily modelled as lagged (as opposed to same‐time) relationships (36 of 63; 57.1%). The time‐lag between EMAs was hourly (63 of 63; 100%), with the number of hours between EMAs ranging from 1 to 8 (median = 4 hours). Across the 63 effect sizes, a median of one additional within‐person predictor (range = 0–3; e.g. coffee consumption), no temporal variables (range = 0–2; e.g. study day), one baseline variable (range = 0–6; e.g. age) and no interaction terms (range = 0–4; e.g. age by negative affect) were included in the statistical models. Most of the effect sizes were estimated using statistical models which included a random intercept (57 of 63; 90.5%), with almost half also including a random slope for the psychological or contextual within‐person predictor (30 of 63; 47.6%). Most studies did not report having centred the psychological or contextual within‐person predictor (34 of 63; 53.9%). Most studies did not report disaggregating predictor–lapse associations into between‐ and within‐person effects (47 of 63; 74.6%).

### Narrative synthesis of predictor–smoking lapse incidence associations

Across eight effect sizes, a negative relationship between behavioural regulation (e.g. cognitive coping, behavioural coping and resisting urges) and lapse incidence was observed for seven effect sizes, with one indicating a positive relationship. Across three effect sizes, a negative relationship between motivation not to smoke (e.g. intention and motivation to quit) and lapse incidence was observed for two effect sizes, with one indicating a positive relationship. Across three effect sizes, a negative relationship between beliefs about capabilities (e.g. self‐efficacy and confidence) and lapse incidence was observed for all effect sizes. Across three effect sizes, a positive relationship between positive feeling states (e.g. positive affect and feeling playful) and lapse incidence was observed for two effect sizes, with one indicating a negative relationship. Across two effect sizes, a positive relationship between positive outcome expectancies (e.g. smoking expectancies) and lapse incidence was observed.

### Meta‐analyses of predictor–smoking lapse incidence associations

#### Negative feeling states

**FIGURE 3:**
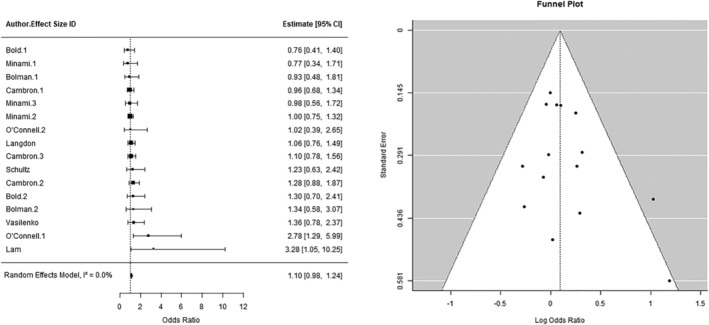
(a) The relationship between negative feeling states and lapse incidence. (b) Funnel plot of studies examining the relationship between negative feeling states and lapse incidence.

A two‐level, random‐effects meta‐analysis (*k* = 16) indicated a non‐significant, positive relationship between negative feeling states (e.g. stress, sadness or anger) and lapse incidence [odds ratio (OR) = 1.10, 95% confidence interval (CI) = 0.98, 1.24, *P* = 0.12; see Figure 3a]. The total between‐study heterogeneity was low (*I* ^2^ = 0.006%). In the planned sensitivity analysis with robust variance estimation, there was a significant, positive relationship between negative feeling states and lapse incidence (OR = 1.12, 95% CI = 1.02, 1.23, *P* = 0.02). There was some evidence of funnel plot asymmetry (see Figure 3b); however, Egger’s test was not significant (*P* = 0.11). Due to the low between‐study heterogeneity, we opted not to go ahead with the moderator analysis.

#### Environmental and social cues

**FIGURE 4:**
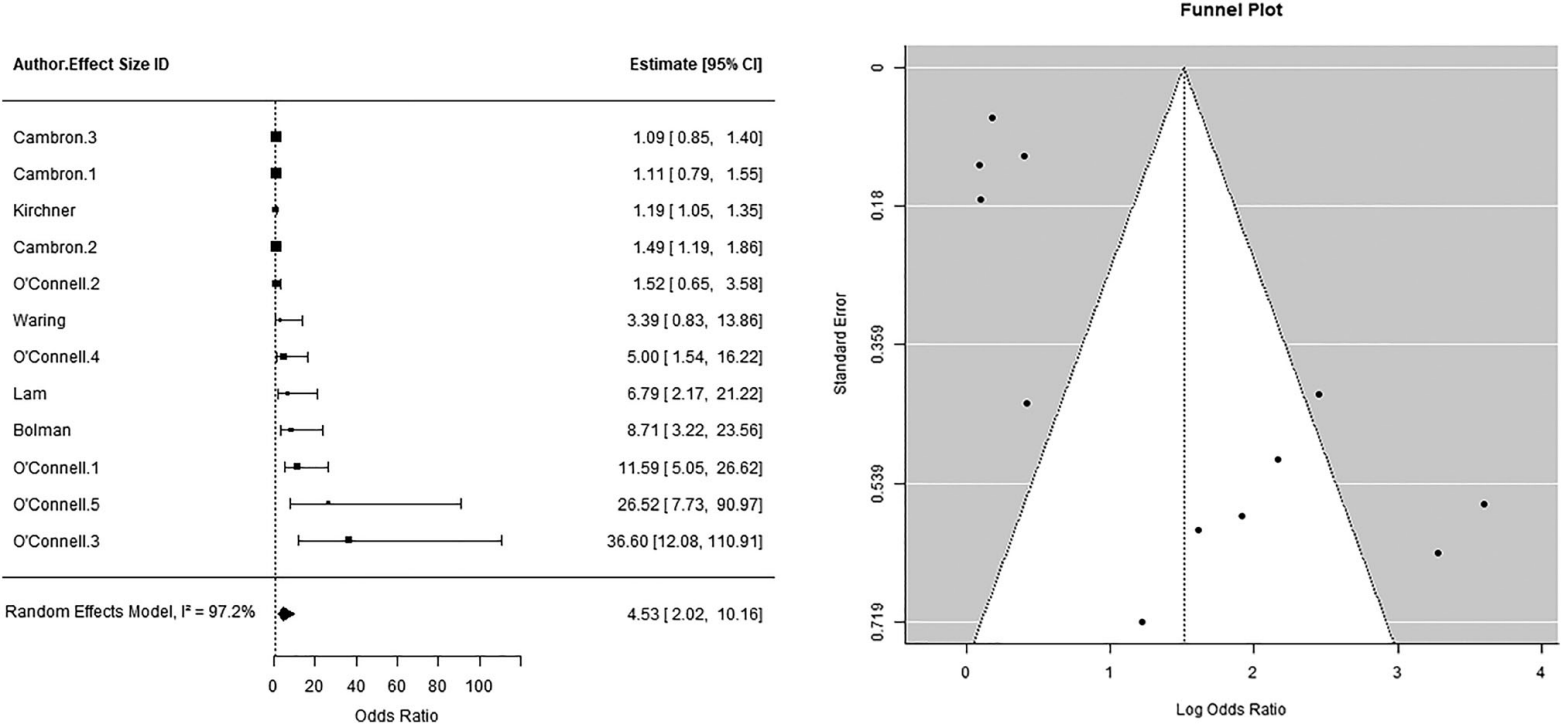
(a) The relationship between environmental and social cues and lapse incidence. (b) Funnel plot of studies examining the relationship between environmental and social cues and lapse incidence.

A three‐level, random‐effects meta‐analysis (*k* = 12) found a significant, positive relationship between environmental and social cues (e.g. cigarette availability or the presence of other smokers) and lapse incidence (OR = 4.53, 95% CI = 2.02, 10.16, *P* = 0.001; see Figure 4a). The total between‐study heterogeneity (level 2 = 41.9% and level 3 = 55.3%) was high (*I* ^2^ = 97.2%). In an unplanned sensitivity analysis to examine the influence of the very large effect sizes in the studies by O’Connell *et al*., the pooled effect attenuated when these effect sizes were excluded (OR = 2.74, 95% CI = 1.16, 6.47, *P* = 0.02). In the sensitivity analysis with robust variance estimation, there was a significant, positive relationship between environmental and social cues and lapse incidence, but the CI widened (OR = 4.31, 95% CI = 1.58, 11.8, *P* = 0.01). There was evidence of funnel plot asymmetry (see Figure 4b) and Egger’s test was significant (*P* < 0.001). In the planned moderator analysis, none of the included moderator variables was significantly associated with the observed outcome (all *P*s > 0.05; see the Supporting information). The inclusion of moderators only marginally reduced the total between‐study heterogeneity (*I* ^2^ = 94.9%).

#### Cravings

**FIGURE 5:**
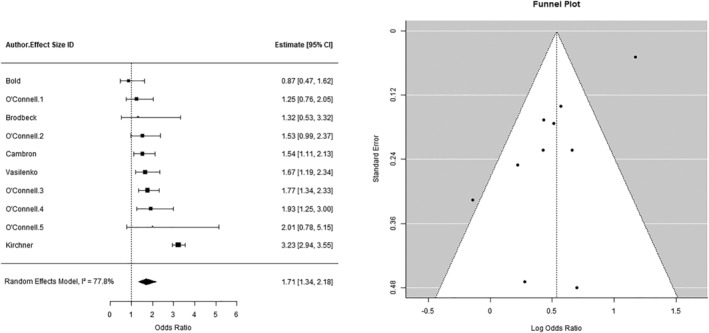
(a) The relationship between cravings and lapse incidence. (b) Funnel plot of studies examining the relationship between cravings and lapse incidence.

A three‐level, random‐effects meta‐analysis (*k* = 10) found a significant, positive relationship between cravings and lapse incidence (OR = 1.71, 95% CI = 1.34, 2.18, *P* < 0.001; see Figure 5a). The total between‐study heterogeneity (level 2 = 38.9% and level 3 = 38.9%) was high (*I* ^2^ = 77.8%). In the sensitivity analysis with robust variance estimation, results remained largely unchanged (OR = 1.67, 95% CI = 1.28, 2.18, *P* = 0.002). There was some evidence of funnel plot asymmetry (see Figure 5b); however, Egger’s test was not significant (*P* = 0.22). In the moderator analysis, studies with participants with a greater mean age (OR = 0.60, 95% CI = 0.43–0.83, *P* < 0.01) and where participants were provided with a flat payment as incentive (OR = 0.00, 95% CI = 0.00–0.004, *P* < 0.01) were associated with significantly smaller effects. Studies with a greater percentage identifying as white ethnicity (OR = 1.03, 95% CI = 1.01–1.05, *P* < 0.001) were associated with significantly larger effects (see the Supporting information). The inclusion of moderators removed the total between‐study heterogeneity (*I* ^2^ = 0%).

## DISCUSSION

This systematic review and meta‐analysis synthesized within‐person associations of psychological and contextual factors with lapse incidence in healthy adult smokers attempting to quit. In addition, it summarized how EMA researchers have conceptualized ‘lapse’ and ‘relapse’, the theoretical underpinning of EMA study designs and the proportion of studies drawing on Open Science principles.

### Within‐person predictor–smoking lapse associations

In meta‐analyses, negative feeling states (e.g. stress and sadness) did not show consistent significant positive associations with lapse incidence. Environmental and contextual cues, as well as cravings, however, were significantly positively associated with lapse incidence, although as few studies reported conducting an a priori power analysis (quality 2) or declared how EMA missingness was treated in the statistical models (quality 4), the confidence in these estimates is reduced. Although we did not pre‐register a smallest effect size of interest prior to conducting the meta‐analyses, following inspection of the results and with a view to informing future studies we would argue that even a 10% increase in the odds of lapsing when encountering a particular cue would be considered clinically meaningful. Even a single lapse could, in some circumstances, be the end of a quit attempt, with the person rapidly returning to smoking as regular. Other psychological and contextual lapse predictors were less frequently examined, with a narrative synthesis indicating negative relationships between behavioural regulation, motivation not to smoke and beliefs about capabilities and lapse incidence, and positive relationships between positive feeling states and positive outcome expectations and lapse incidence.

The finding that negative feeling states (e.g. stress or sadness) did not show consistent significant positive associations with lapse incidence merits further thought. This finding is consistent with recent meta‐analytical findings from the alcohol consumption field, indicating that people are more likely to drink (or drink more heavily) on days when they experience higher than typical positive but not negative affect [43]. Conversely, the absence of a significant association may be an artefact of several methodological aspects. First, it is plausible that high‐ rather than low‐arousal negative affect is a key driver of lapses—i.e. anxiety or anger as opposed to sadness. Instruments frequently used to capture affect in EMA studies (e.g. the Positive and Negative Affect Schedule; PANAS) tend to explicitly include high‐arousal negative affect items (e.g. angry and upset) but treat low scores on the positive affect items as indicative of low‐arousal negative affect (e.g. sad and lethargic). As researchers often supplement PANAS items with items specifically capturing low‐arousal negative affect in EMA studies, and combine these into general negative and positive affect subscales, it is plausible that the lack of discrimination between high‐ and low‐arousal negative affect in our meta‐analysis is driving the non‐significant association. We did not code the instruments used to capture negative affect here, but such granular investigation merits further exploration in future research. Second, it is plausible that negative affect exhibits high temporal instability and is therefore particularly susceptible to the time‐lag between EMA prompts (discussed in more detail below). Any significant association between negative feeling states and lapse incidence may be moderated by the time‐lag selected—this also merits further investigation. Third, the finding that negative feeling states was not consistently positively associated with lapse incidence may reflect the mixed‐effects modelling approach used in the included studies, which require within‐person associations to be consistently observed across individuals to be detected. However, evidence indicates that different predictor variables are important for different individuals (i.e. lapse incidence is ‘idiosyncratic’). Fourth, the finding that environmental and contextual cues are strongly associated with the risk of lapsing (consistent with prior research [7, 10]) may also provide an alternative explanation for this non‐significant association (discussed further below), as it may be interpreted to suggest that opportunity (e.g. cigarette availability) is vital for lapses to occur, irrespective of what triggered the desire to smoke.

Coupled with our second key result, that environmental and contextual cues are strongly associated with the risk of lapsing, it is plausible that negative feeling states may have initially triggered strong cravings but not materialized if there was no opportunity to act. It should be noted that many of the effect sizes extracted from the included studies were estimated using multivariable (adjusted) models—any effect of negative feeling states may therefore have been suppressed when taking account of cravings and environmental variables for which there may be a stronger link with lapse incidence. A clearer understanding of the causal chain of events—e.g. negative affect triggering a craving, which leads to the person seeking out cigarettes vs. exposure to someone smoking in one’s immediate environment triggering negative affect and cravings, which then leads the person to smoke—is required. Available statistical modelling approaches (e.g. multi‐level models, including multi‐level mediation models) are not ideal for examining such complex causal chains. This merits further investigation using computational modelling techniques which can take account of the dynamic and multi‐factorial nature of lapse incidence, such as dynamic systems modelling [44, 45]. Such work is currently being undertaken in project COMPLAPSE, led by the first author and funded by the European Commission (https://www.olgaperski.com/research/complapse.html).

### Definitions of ‘lapse’ and ‘relapse’

Although lapse definitions were comparable, relapse definitions varied widely across studies. Most commonly, ‘threshold’ definitions of relapse were used. Although it is positive that there is consistency in how lapses have been defined in the EMA literature—with definitions corresponding largely to how lapses have been defined in between‐person studies, including clinical trials—the variability in relapse definitions poses several challenges for the EMA and smoking cessation research communities. First, many definitions (e.g. ‘return to regular smoking’) appear too imprecise to be useful in clinical trials or to underpin EMA studies. Conversely, threshold definitions risk being arbitrary. Further research linking threshold definitions and associated patterns of smoking with longer‐term abstinence are therefore needed, examining the sensitivity and specificity of different cut‐offs. Novel data collection methods, including EMA and passive sensor data, may allow a reconceptualization of relapse. With individuals monitored regularly over longer time‐frames, it may be possible to empirically determine smoking patterns during a quit attempt which are indicative of relapse both within and between individuals. Similar to recent work on the conceptualization and operationalization of physical activity maintenance [46], further work—harnessing EMAs—is required to generate a better understanding of smoking relapse.

### Theoretical underpinning of EMA designs

The finding that few studies explicitly drew upon psychological theory to inform EMA study design decisions beyond what psychological or contextual variables to examine (e.g. sampling frequency and study duration) was not unexpected. As emphasized by other scholars, many available psychological theories lack information regarding the expected temporal dynamics of psychological processes [47]. However, of the studies which mentioned theory, the most commonly used one was Relapse Prevention Theory—an influential theory within the smoking cessation and addiction domain which has itself been revised using evidence from EMA studies [14]. The most frequently used time‐lag between EMAs in the included studies was 4 hours, which was not informed by any theory. It is plausible that negative feeling states, cravings, etc. change at a faster rate than 4 hours, with peaks in such constructs conferring imminent lapse risk. For example, evidence indicates that lapses occur within 11 minutes following exposure to particular psychological or contextual cues [17]. Important signals that the person is at risk of lapsing may therefore remain undetected based on current EMA study designs. However, capturing psychological and contextual variables at their appropriate temporal granularity also needs to be carefully balanced with participant burden and the risk of negatively influencing EMA adherence.

### Open Science principles

The finding that only one of the included studies drew upon Open Science principles of pre‐registration and data sharing may be due to the time span during which studies were published (i.e. 1996–2022, with the majority published before 2015), during which Open Science had not yet started to proliferate (e.g. the not‐for‐profit organization ‘Centre for Open Science’ was founded in 2013; https://www.cos.io/). As argued elsewhere, we strongly encourage the use of Open Science principles in EMA research [29].

### Strengths

First, this review was conducted by an international and versatile team of researchers with expertise spanning smoking cessation, health psychology and EMA research. Second, this was the first study to synthesize momentary antecedents of lapse incidence in smokers attempting to stop. Third, this review drew upon principles of Open Science, including review pre‐registration; documentation of design and analytical decisions; and the sharing of analytical code and data for transparency and to enable re‐use [48]. We strongly encourage other EMA researchers to use and update the electronic searches and the database of EMA smoking lapse studies.

### Limitations

First, due to the review scope (i.e. adult and non‐clinical populations), the results may not generalize to adolescent smokers or smokers with physical or mental health problems. Second, as the items used to assess the psychological/contextual variables differed with regard to the number of response options (e.g. five‐point scales, seven‐point scales or presence vs. absence) and time‐scales addressed (e.g. ‘right now’ and ‘since the last assessment’), and different EMA time‐lags were used (e.g. every 4 hours and twice per day), the interpretation of the meta‐analytical results is not entirely straightforward. Future work should consider converting EMA item scores to the percentage of maximum possible (POMP) score [43] prior to pooling the results and/or using a continuous‐time meta‐analytical approach [49]; however, this requires individual‐level data to be obtained from study authors. Given the scope of the larger review and limited resource, we were unable to consider these approaches in the current review. Differences in item response options and time‐lags may therefore have influenced the results. Third, although we examined whether the included studies drew upon Open Science principles (i.e. pre‐registration of study protocols and data sharing), we did not assess the quality of implementation. This turned out not to be an issue for the present review, as only one of the included studies met our basic threshold (i.e. ‘yes’ vs. ‘no’) detected by assessing whether the included studies mentioned/linked to a pre‐registration or data repository anywhere in the article. Fourth, there was an insufficient number of studies to examine within‐level interactions between psychological/contextual variables or cross‐level interactions between more stable traits/factors measured at baseline (e.g. personality and nicotine dependence) and EMA‐assessed psychological/contextual variables. Related to the previous point, due to the small number of studies that could be included in the moderator analyses, estimates were unreliable and need to be interpreted with much caution. Future review work with a larger number of included studies would benefit from including additional moderator variables (e.g. the EMA time‐lag and the specific psychological constructs assessed, as opposed to the larger groupings used here). Fifth, we conducted an unplanned sensitivity analysis to examine the influence of the very large effect sizes in the studies by O’Connell *et al*. pertaining to the association of environmental and social cues and lapse incidence. However, it may be argued that effect sizes of such magnitude (e.g. those pertaining to a tenfold or larger increase in the odds to lapse incidence) are implausible and should be excluded from future meta‐analyses. Similar to the above discussion about a ‘smallest effect size of interest’, it may be fruitful for researchers to also consider an *a priori* ‘largest plausible effect size of interest’ and use this to inform the analyses. Finally, the selected inclusion and exclusion criteria inevitably narrowed both the range of studies included in the systematic review and the effect sizes contributing to the meta‐analyses. For example, given the focus on within‐person predictor–lapse associations, we did not include effect sizes in the meta‐analyses pertaining to EMA‐assessed predictor–lapse associations that had been estimated at the between‐subjects level (i.e. marginal models) using, for example, generalized estimating equations or survival analysis, with the latter also introducing the non‐trivial issue of converting hazard ratios (HRs) to ORs prior to pooling. Future work should consider contacting authors to access the raw data (which could be enabled by Open Science practices being used more widely) to maximize the number of effect sizes available for meta‐analysis.

### Wider implications and avenues for future research

This review strengthens existing evidence highlighting environmental and social cues as substantial drivers of smoking lapse. More work is needed to more clearly understand how their influence can be ameliorated. In addition to strengthening policies which limit the availability and affordability of cigarettes, just‐in‐time adaptive interventions (JITAIs) may be particularly useful for pre‐empting moments of lapse risk and encouraging smokers to try behavioural substitution (e.g. drinking a glass of water), distraction or even removing themselves from potentially ‘dangerous’ situations [20]. We also need further research to explore approaches for rapidly altering motivation to extinguish strong cravings to smoke.

We note that none of the included studies drew upon advancements in sensor technology to passively detect smoking lapses and/or psychological or contextual predictors (e.g. digital biomarkers such as heart rate variability or weather). For the past decade, it has been argued that it would be useful for passive sensor data streams to be harnessed to predict high‐risk smoking situations and support the delivery of real‐time support, which would increase the temporal granularity of assessments and reduce participant burden [50]. Therefore, this remains an important avenue for future research.

EMA studies enable researchers to test psychological theory within individuals over time and build dynamic behaviour change theories. However, few studies included in the present review explicitly tested or aimed to build dynamic theories. Further theoretical work is needed—drawing upon the rich data from EMA studies—to refine available psychological theories to more effectively account for the dynamic nature of smoking lapse risk.

Finally, future reviews should go beyond considering only the presence versus absence of Open Science principles. For example, EMA researchers may provide a pre‐registered study protocol and analysis plan, but not specify the many statistical decisions that need to be made (e.g. the inclusion of random intercepts and slopes for the within‐person predictors and adjustment for temporal variables).

## CONCLUSION

This systematic review and meta‐analysis synthesized within‐person psychological and contextual predictor–lapse associations among smokers attempting to quit. Environmental and social cues and cravings are key within‐person predictors of lapse incidence during a quit attempt although, due to low study quality, the confidence in these estimates is reduced. In addition, we examined how EMA researchers have defined ‘lapse’ and ‘relapse’, summarized the theoretical underpinning of EMA study designs and summarized the proportion of studies with pre‐registered study protocols/analysis plans and open data. Although lapse definitions were comparable, relapse definitions varied widely across studies. Few studies explicitly drew upon psychological theory to inform EMA study design decisions. One of the included studies drew upon Open Science principles of pre‐registration or data sharing.

## AUTHOR CONTRIBUTIONS

**Olga Perski:** Conceptualization (lead); data curation (lead); methodology (lead); writing ‐ original draft (lead); writing ‐ review and editing (lead). **Dominika Kwasnicka:** Conceptualization (lead); data curation (equal); methodology (lead); writing—review and editing (equal). **Dimitra Kale:** Conceptualization (equal); data curation (equal); writing—review and editing (equal). **Verena Schneider:** Conceptualization (equal); data curation (equal); writing—review and editing (equal). **Dorothy Szinay:** Conceptualization (equal); data curation (equal); writing—review and editing (equal). **Gill ten Hoor:** Conceptualization (equal); data curation (equal); writing—review and editing (equal). **Bernard Yeboah‐Asiamah Asare:** Conceptualization (equal); data curation (equal); writing—review and editing (equal). **Peter Verboon:** Conceptualization (equal); methodology (equal); writing—review and editing (equal). **Daniel Powell:** Conceptualization (equal); methodology (equal); writing—review and editing (equal). **Felix Naughton:** Conceptualization (equal); methodology (equal); writing—review and editing (equal). **Jan Keller:** Conceptualization (equal); data curation (equal); methodology (equal); writing—review and editing (equal).

## DECLARATION OF INTERESTS

The authors have no conflicts of interest to declare.

## Supporting information


**Data S1.** Supporting information.

## Data Availability

The data and R code underpinning the analyses are available via GitHub (https://github.com/OlgaPerski/EMA_smoking_lapse_review).
