# Supplementary material for: Within‐person associations between psychological and contextual factors and lapse incidence in smokers attempting to quit: A systematic review and meta‐analysis of ecological momentary assessment studies
Source: Addiction. 2023 Mar 10;118(7):1216–31. doi: 10.1111/add.16173 (PMC10952786; doi:10.1111/add.16173)
Supplement: Supplementary file 1 — Data S1. Supporting information. [file ADD-118-1216-s001.docx]

**Supplementary Materials**

***Initial search strategy (January 2020 and February 2021)***

***Ovid MEDLINE, Embase, PsycINFO***

1. (ecological adj1 momentary adj1 assessment*).ti,ab.

2. (intensive adj1 longitudinal).ti,ab.

3. (ambulatory adj1 assessment*).ti,ab.

4. (experience adj1 sampl*).ti,ab.

5. (daily adj1 diar*).ti,ab.

6. (ecological adj1 momentary adj1 intervention).ti,ab.

7. within-person.ti,ab.

8. within-subject*.ti,ab.

9. (single adj1 case).ti,ab.

10. idiographic.ti,ab.

11. 1 OR 2 OR 3 OR 4 OR 5 OR 6 OR 7 OR 8 OR 9 OR 10

12. tobacco.ti,ab.

13. smok*.ti,ab.

14. alcohol*.ti,ab.

15. diet.ti,ab.

16. weight.ti,ab.

17. overweight.ti,ab.

18. obes*.ti,ab.

19. (healthy adj3 eat*).ti,ab.

20. physical activity.ti,ab.

21. exercise.ti,ab.

22. (medication adj1 adherence).ti,ab.

23. (treatment adj1 adherence).ti,ab.

24. (sexual adj1 health).ti,ab.

25. condom.ti,ab.

26. contraceptive.ti,ab.

27. 12 OR 13 OR 14 OR 15 OR 16 OR 17 OR 18 OR 19 OR 20 OR 21 OR 22 OR 23 OR 24 OR 25 OR 26

28. 11 AND 27

***Web of Science***

1. TS=(ecological NEAR/1 momentary NEAR/1 assessment*)

2. TS=(ecological NEAR/1 momentary NEAR/1 intervention)

3. TS=(intensive NEAR/1 longitudinal)

4. TS=(ambulatory NEAR/1 assessment*)

5. TS=(experience NEAR/1 sampl*)

6. TS=(daily NEAR/1 diar*)

7. TS=(within-person or within-subject* or idiographic)

8. TS=(single NEAR/1 case)

9. 1 OR 2 OR 3 OR 4 OR 5 OR 6 OR 7 OR 8

10. TS=(healthy NEAR/1 eat*)

11. TS=(medication NEAR/1 adherence)

12. TS=(treatment NEAR/1 adherence)

13. TS=(sexual NEAR/1 health)

14. TS=(smok* or tobacco* or alcohol* or diet or weight or overweight or obes* or physical activity or exercise or condom or contraceptive)

15. 10 OR 11 OR 12 OR 13 OR 14

16. 9 AND 15

***Updated search strategy (November 2022)***

***Ovid MEDLINE, Embase, PsycINFO***

1. (ecological adj1 momentary adj1 assessment*).ti,ab.

2. (intensive adj1 longitudinal).ti,ab.

3. (ambulatory adj1 assessment*).ti,ab.

4. (experience adj1 sampl*).ti,ab.

5. (daily adj1 diar*).ti,ab.

6. (ecological adj1 momentary adj1 intervention).ti,ab.

7. within-person.ti,ab.

8. within-subject*.ti,ab.

9. (single adj1 case).ti,ab.

10. idiographic.ti,ab.

11. electronic diar*.ti,ab.

12. real-time.ti,ab.

13. mobile device*.ti,ab.

14. handheld device*.ti,ab.

15. palmtop.ti,ab.

16. day-to-day.ti,ab.

17. 1 OR 2 OR 3 OR 4 OR 5 OR 6 OR 7 OR 8 OR 9 OR 10 OR 11 OR 12 OR 13 OR 14 OR 15 OR 16

18. tobacco.ti,ab.

19. smok*.ti,ab.

20. 18 OR 19

21. 17 AND 20

***Web of Science***

1. TS=(ecological NEAR/1 momentary NEAR/1 assessment*)

2. TS=(ecological NEAR/1 momentary NEAR/1 intervention)

3. TS=(intensive NEAR/1 longitudinal)

4. TS=(ambulatory NEAR/1 assessment*)

5. TS=(experience NEAR/1 sampl*)

6. TS=(daily NEAR/1 diar*)

7. TS=(within-person or within-subject* or idiographic)

8. TS=(single NEAR/1 case)

9. TS=(electronic diar*)

10. TS=(real-time)

11. TS=(mobile device*)

12. TS=(handheld device*)

13. TS=(palmtop)

14. TS=(day-to-day)

15. TS=(smok* or tobacco*)

16. 1 OR 2 OR 3 OR 4 OR 5 OR 6 OR 7 OR 8 OR 9 OR 10 OR 11 OR 12 OR 13 OR 14

17. 15 AND 16

***Data extraction form***

- *Study description:* lead author, year, country, setting;
- *Participant characteristics:* sample size, mean age (SD), gender (% female), educational attainment (% university education), ethnicity (% White ethnicity);
- *Baseline smoking characteristics:* mean cigarettes per day (SD), mean number of past quit attempts (SD), mean level of motivation to stop (SD), current use of cessation aids (% used pharmacotherapy, % used nicotine replacement therapy, % used behavioural support);
- *Lapse incidence:* measurement type (e.g., self-report, carbon monoxide monitor);
- *Psychological/contextual predictors:* construct(s) assessed (e.g., craving, positive affect, negative affect, self-efficacy), measurement type (e.g., self-report, wearable device), whether a single- or multi-item instrument was used, whether there was a precedent for the instrument used;
- *Study type:* observational, interventional;
- *EMA delivery mode:* mobile phone, smartphone, hand-held device, pen-and-paper;
- *EMA method:* signal-contingent, event-contingent, multiple;
- *Study duration in days*;
- *Frequency of EMAs:* daily, multiple times per day, weekly.
- *Authors’ definition of ‘lapse’ and ‘relapse’*;
- *Theoretical underpinning of the EMA study design*;
- *Whether the study design and/or analysis plan had been pre-registered*;
- *Whether the data underpinning the analyses had been made openly available to other researchers via a public repository*;
- *Type of statistical model used:* e.g., hierarchical/multilevel model, multilevel structural equation model;
- *Treatment of missing data:* e.g., restricted maximum likelihood (REML), full information maximum likelihood (FIML), multiple imputation (MI);
- *Number of observations used for the within-person analysis*;
- *Number and type of within-person psychological or contextual predictor(s)*;
- *Whether the modelled relationship between the predictor and outcome variables pertains to a same-time or lagged relationship (if the latter, the time lag was coded)*;
- *Additional within-person, between-person, interaction terms, and temporal (e.g., days in study) predictors/covariates included in the model*;
- *Whether a random intercept and/or random slope for the within-person psychological/contextual predictor(s) were used*;
- *Whether within- and between-person effects were disaggregated (i.e., by simultaneously modelling the within- and between-person effects of the psychological/contextual predictor)*;
- *Coefficients (e.g., odds ratios, risk ratios) and standard errors for the within- and between-person associations between the psychological/contextual predictor(s) and the smoking lapse outcome*.

***Psychological and contextual predictors***

| **ID** | **Author** | **Year** | **Author psychological/contextual predictor** | **Psychological/contextual predictor coding** |
| --- | --- | --- | --- | --- |
| 4_1 | Allen | 2008 | agitation | negative feeling states |
| 46_1 | Bold | 2016 | distress | negative feeling states |
| 46_2 | Bold | 2016 | urge | cravings |
| 46_3 | Bold | 2016 | alcohol | other |
| 46_4 | Bold | 2016 | not suitable for meta-analysis | NA |
| 47_1 | Bold | 2016 | self-efficacy | beliefs about capabilities |
| 48_1 | Bolman | 2018 | activity dissatisfaction | negative feeling states |
| 48_2 | Bolman | 2018 | positive outcome expectations | positive outcome expectations |
| 48_3 | Bolman | 2018 | negative affect | negative feeling states |
| 48_4 | Bolman | 2018 | being around other smokers | environmental and social cues |
| 48_5 | Bolman | 2018 | urge | cravings |
| 60_1 | Brodbeck | 2013 | not suitable for meta-analysis | NA |
| 61_1 | Brodbeck | 2014 | not suitable for meta-analysis | NA |
| 68_1 | Businelle | 2016 | stress | negative feeling states |
| 72_1 | Cambron | 2019 | negative affect | negative feeling states |
| 72_2 | Cambron | 2019 | urge | cravings |
| 72_3 | Cambron | 2019 | self-efficacy | beliefs about capabilities |
| 72_4 | Cambron | 2019 | motivation to stop | motivation not to smoke |
| 72_5 | Cambron | 2019 | difficulty concentrating | memory, attention and decision processes |
| 72_6 | Cambron | 2019 | coping expectancies | other |
| 72_7 | Cambron | 2019 | smoking expectancies | positive outcome expectations |
| 72_8 | Cambron | 2019 | other smokers present | environmental and social cues |
| 73_1 | Cambron | 2020 | cigarette availability | environmental and social cues |
| 73_2 | Cambron | 2020 | smoking allowed | environmental and social cues |
| 73_3 | Cambron | 2020 | not suitable for meta-analysis | NA |
| 87_1 | Catley | 2000 | not suitable for meta-analysis | NA |
| 97_1 | Cofta-Woerpel | 2011 | not suitable for meta-analysis | NA |
| 122_1 | Dermody | 2020 | not suitable for meta-analysis | NA |
| 123_1 | Derrick | 2018 | not suitable for meta-analysis | NA |
| 151_1 | Etcheverry | 2016 | not suitable for meta-analysis | NA |
| 158_1 | Ferguson | 2006 | not suitable for meta-analysis | NA |
| 588_1 | Ferguson & Shiffman | 2010 | not suitable for meta-analysis | NA |
| 205_1 | Gwaltney | 2005a | not suitable for meta-analysis | NA |
| 206_1 | Gwaltney | 2005b | not suitable for meta-analysis | NA |
| 589_1 | Gwaltney | 2002 | exposure to point-of-sale tobacco | environmental and social cues |
| 263_1 | Kirchner | 2013 | craving | cravings |
| 263_2 | Kirchner | 2013 | not suitable for meta-analysis | NA |
| 267_1 | Koslovsky | 2018 | alcohol consumption | other |
| 270_1 | Lam | 2014 | negative affect | negative feeling states |
| 270_2 | Lam | 2014 | presence of others smoking | environmental and social cues |
| 270_3 | Lam | 2014 | negative affect | negative feeling states |
| 273_1 | Langdon | 2016 | not suitable for meta-analysis | NA |
| 295_1 | Liu | 2013 | not suitable for meta-analysis | NA |
| 331_1 | McCarthy | 2008 | not suitable for meta-analysis | NA |
| 332_1 | McCarthy | 2010 | not suitable for meta-analysis | NA |
| 592_1 | McCarthy | 2006 | not suitable for meta-analysis | NA |
| 343_1 | Messer | 2018 | not suitable for meta-analysis | NA |
| 345_1 | Minami | 2017 | stressful event | negative feeling states |
| 346_1 | Minami | 2010 | coping | behavioural regulation |
| 346_2 | Minami | 2010 | negative affect | negative feeling states |
| 346_3 | Minami | 2010 | positive affect | positive feeling states |
| 346_4 | Minami | 2010 | not suitable for meta-analysis | NA |
| 347_1 | Minami | 2015 | negative affect | negative feeling states |
| 348_1 | Minami | 2014 | positive affect | positive feeling states |
| 348_2 | Minami | 2014 | confidence | beliefs about capabilities |
| 348_3 | Minami | 2014 | willingness to work | motivation not to smoke |
| 348_4 | Minami | 2014 | not suitable for meta-analysis | NA |
| 373_1 | O'Connell | 2000 | not suitable for meta-analysis | NA |
| 374_1 | O'Connell | 2002 | number of coping strategies used | behavioural regulation |
| 375_1 | O'Connell | 2007 | intention to implement a coping strategy | motivation not to smoke |
| 375_2 | O'Connell | 2007 | number of resisted urges | behavioural regulation |
| 376a_1 | O'Connell | 2008 | urge | cravings |
| 376a_2 | O'Connell | 2008 | number of resisted urges | behavioural regulation |
| 376b_1 | O'Connell | 2008 | urge | cravings |
| 376b_2 | O'Connell | 2008 | cigarette availability | environmental and social cues |
| 377a_1 | O'Connell | 2010 | others smoking | environmental and social cues |
| 377a_2 | O'Connell | 2010 | craving | cravings |
| 377a_3 | O'Connell | 2010 | cigarette availability | environmental and social cues |
| 377b_1 | O'Connell | 2010 | others smoking | environmental and social cues |
| 377b_2 | O'Connell | 2010 | craving | cravings |
| 377b_3 | O'Connell | 2010 | playful state | positive feeling states |
| 594_1 | O’Connell | 2004 | rebellious state | negative feeling states |
| 594_2 | O’Connell | 2004 | negative affect | negative feeling states |
| 594_3 | O’Connell | 2004 | cigarette availability | environmental and social cues |
| 594_4 | O’Connell | 2004 | urge | cravings |
| 594_5 | O’Connell | 2004 | coping | behavioural regulation |
| 594_6 | O’Connell | 2004 | not suitable for meta-analysis | NA |
| 405_1 | Peters | 2009 | not suitable for meta-analysis | NA |
| 472_1 | Shiffman | 2007 | not suitable for meta-analysis | NA |
| 480_1 | Shiffman | 2008 | not suitable for meta-analysis | NA |
| 485_1 | Shiffman | 1996 | not suitable for meta-analysis | NA |
| 509_1 | Spears | 2019 | not suitable for meta-analysis | NA |
| 520_1 | Suchting | 2019 | urge | cravings |
| 549_1 | Vasilenko | 2014 | negative affect | negative feeling states |
| 549_2 | Vasilenko | 2014 | not suitable for meta-analysis | NA |
| 551_1 | Vinci | 2017 | offered a cigarette | environmental and social cues |
| 558_1 | Waring | 2020 | supported to quit | positive social support |
| 558_2 | Waring | 2020 | pressured to quit | negative social support |
| 558_3 | Waring | 2020 | not suitable for meta-analysis | NA |
| 578_1 | Yeh | 2012 | not suitable for meta-analysis | NA |
| 617_1 | Buitenhuis | 2021 | stress | negative feeling states |
| 620_1 | Cambron | 2020 | not suitable for meta-analysis | NA |
| 655_1 | Hebert | 2021 | not suitable for meta-analysis | NA |
| 693_1 | Nakajima | 2020 | not suitable for meta-analysis | NA |
| 703_1 | Potter | 2021 | not suitable for meta-analysis | NA |
| 716_1 | Shiffman | 2020 | behavioural coping | behavioural regulation |
| 717_1 | Shiffman | 2020 | cognitive coping | behavioural regulation |
| 717_2 | Shiffman | 2020 | both behavioural and cognitive coping | behavioural regulation |
| 717_3 | Shiffman | 2020 | not suitable for meta-analysis | NA |
| 901_1 | Chakraborti | 2022 | not suitable for meta-analysis |  |
| 909_1 | Liang | 2021 | not suitable for meta-analysis |  |
| 910_1 | Schultz | 2021 | stressful event intensity | negative feeling states |
| 911_1 | Vinci | 2021 | not suitable for meta-analysis |  |
| 913_1 | Majmundar | 2020 | not suitable for meta-analysis |  |
| 918_1 | Shiffman | 1997 | not suitable for meta-analysis |  |

*Note.* NA = Not applicable.

***Definitions of ‘lapse’ and ‘relapse’***

| **Author** | **Year** | **Author lapse definition** | **Lapse coding** | **Author relapse definition** | **Relapse coding** |
| --- | --- | --- | --- | --- | --- |
| Allen | 2008 | A single puff from a cigarette | any smoking after the quit date | A single puff from a cigarette | any smoking after the quit date |
| Bold | 2016 | Any smoking in the past 2 hours or since the last report | any smoking since the last report | Any smoking after the quit date | any smoking after the quit date |
| Bold | 2016 | Whether they smoked in the last 2 hours | any smoking over a defined time frame | NR |  |
| Bolman | 2018 | Any smoking since the last beep | any smoking since the last report request | ≥5 cigarettes on 3-7 consecutive days | threshold |
| Brodbeck | 2013 | Any smoking since filling out the last questionnaire | any smoking since the last report | Abandonment of the abstinence goal | stopped trying |
| Brodbeck | 2014 | NR |  | Abandonment of the abstinence goal | stopped trying |
| Businelle | 2016 | Smoking even a puff after the quit date | any smoking after the quit date | Smoking even a puff after the quit date | any smoking after the quit date |
| Cambron | 2019 | Whether they had smoked any cigarettes | any smoking after the quit date | Return to regular smoking | undefined regular smoking |
| Cambron | 2020 | Whether they had smoked any cigarettes | any smoking after the quit date | NR |  |
| Catley | 2000 | NR |  | No longer trying to quit smoking | stopped trying |
| Cofta-Woerpel | 2011 | specific incident of smoking at any time during the quit attempt | any smoking after the quit date | Any smoking (i.e. even a puff) during the period indicated in the point prevalence abstinence measure | any smoking after the quit date |
| Dermody | 2020 | any time they smoked, even if it was just one puff | any smoking after the quit date | Smoking at least 5 cigarettes per day for 3 consecutive days | threshold |
| Derrick | 2018 | NR |  | Five or more lapses at 21 days | threshold |
| Etcheverry | 2016 | NR |  | Any smoking after the quit date | any smoking after the quit date |
| Ferguson | 2006 | NR |  | NR |  |
| Ferguson & Shiffman | 2010 | individual instances of smoking | any smoking after the quit date | Smoking five cigarettes a day for 3 consecutive days | threshold |
| Gwaltney | 2005a | discrete episodes of smoking | any smoking after the quit date | Smoking five cigarettes a day for 3 consecutive days | threshold |
| Gwaltney | 2005b | a circumscribed smoking episode after complete abstinence | any smoking after the quit date | Smoking five cigarettes a day for 3 consecutive days | threshold |
| Gwaltney | 2002 | any occasion of smoking, even if only a puff | any smoking after the quit date | Smoking five cigarettes a day for 3 consecutive days | threshold |
| Kirchner | 2013 | whether they had smoked in the preceding 2 hours | any smoking over a defined time frame | No longer trying to refrain from use | stopped trying |
| Koslovsky | 2018 | NR |  | NR |  |
| Lam | 2014 | isolated incidents of cigarette smoking | any smoking after the quit date | NR |  |
| Langdon | 2016 | NR |  | NR |  |
| Liu | 2013 | episodes of smoking | any smoking after the quit date | 7 consecutive days of smoking | threshold |
| McCarthy | 2008 | smoked after the quit date | any smoking after the quit date | 7 consecutive days of smoking | threshold |
| McCarthy | 2010 | smoked post-quit | any smoking after the quit date | NR |  |
| McCarthy | 2006 | smoking | any smoking after the quit date | NR |  |
| Messer | 2018 | episodes of smoking during an ongoing quit attempt | any smoking after the quit date | Return to regular smoking | undefined regular smoking |
| Minami | 2017 | any smoking (at least one cigarette) since the last report | smoking at least one cigarette since the last report | NR |  |
| Minami | 2010 | cigarette smoking 48 hours prior to the EMA reporting | any smoking since the last report | NR |  |
| Minami | 2015 | any puff from a cigarette after the target quit day | any smoking after the quit date | 7 consecutive days of smoking | threshold |
| Minami | 2014 | smoking at least one cigarette since last report | smoking at least one cigarette since the last report | Return to regular smoking | undefined regular smoking |
| O'Connell | 2000 | smoking | any smoking after the quit date | Return to regular smoking | undefined regular smoking |
| O'Connell | 2002 | an episode during which any smoking occurred, even a puff | any smoking after the quit date | NR |  |
| O'Connell | 2007 | an episode during which any smoking occurred, even a puff | any smoking after the quit date | ≥5 cigarettes on 3 consecutive days | threshold |
| O'Connell | 2008 | episodes of smoking during a cessation attempt | any smoking after the quit date | ≥5 cigarettes on 3 consecutive days or having a total of 30 lapses | threshold |
| O'Connell | 2008 | episodes of smoking during a cessation attempt | any smoking after the quit date | ≥5 cigarettes on 3 consecutive days or having a total of 30 lapses | threshold |
| O'Connell | 2010 | any smoking, even a puff | any smoking after the quit date | ≥5 cigarettes on 3 consecutive days | threshold |
| O'Connell | 2010 | any smoking, even a puff | any smoking after the quit date | ≥5 cigarettes on 3 consecutive days | threshold |
| O’Connell | 2004 | episodes in which the participant smokes a cigarette | any smoking after the quit date | NR |  |
| Peters | 2009 | smoke one puff of a cigarette | any smoking after the quit date | Return to regular smoking | undefined regular smoking |
| Shiffman | 2007 | NR |  | NR |  |
| Shiffman | 2008 | when the participant started smoking | any smoking after the quit date | ≥5 cigarettes on 3 consecutive days | threshold |
| Shiffman | 1996 | any occasion of smoking, even if only a puff | any smoking after the quit date | Return to regular smoking | undefined regular smoking |
| Spears | 2019 | smoking a cigarette on the quit day | any smoking after the quit date | NR |  |
| Suchting | 2019 | NR |  | NR |  |
| Vasilenko | 2014 | the number of cigarettes smoked since the last occasion | smoking at least one cigarette since the last report | 7 consecutive days of smoking | threshold |
| Vinci | 2017 | smoking a cigarette | any smoking after the quit date | NR |  |
| Waring | 2020 | the number of smoked cigarettes since the last EMA | smoking at least one cigarette since the last report | NR |  |
| Yeh | 2012 | the number of cigarettes smoked since the last report | smoking at least one cigarette since the last report | NR |  |
| Buitenhuis | 2021 | whether they had smoked that day | any smoking after the quit date | Falling back to smoking | undefined regular smoking |
| Cambron | 2020 | one puff of a cigarette or more | any smoking after the quit date | Return to regular smoking | undefined regular smoking |
| Hebert | 2021 | smoked any cigarettes (even a puff) | any smoking after the quit date | NR |  |
| Nakajima | 2020 | a smoking episode | any smoking after the quit date | NR |  |
| Potter | 2021 | whether a participant smoked one or more times in the interval between two EMAs | any smoking since the last report | 7 consecutive days of smoking (SRNT criterion; Piper et al., 2020) | threshold |
| Shiffman | 2020 | NR |  | NR |  |
| Shiffman | 2020 | NR |  | NR |  |
| Chakraborti | 2022 | NR |  | NR |  |
| Liang | 2021 | whether or not a subject reported smoking in the 4 hr prior to the current EMA | any smoking since the last report | NR |  |
| Schultz | 2021 | the number of cigarettes smoked, dichotomised | any smoking after the quit date | NR |  |
| Vinci | 2021 | whether they had smoked any cigarettes | any smoking after the quit date | NR |  |
| Majmundar | 2020 | whether they smoked | any smoking after the quit date | NR |  |
| Shiffman | 1997 | NR |  | NR |  |

*Note.* NR = Not reported.

***Theoretical underpinning of EMA study designs***

| **Author** | **Year** | **Author theory** | **Theory coding** | **Theory informed the study design** | **Theory informed variables assessed** | **Theory informed EMA frequency or timing** |
| --- | --- | --- | --- | --- | --- | --- |
| Allen | 2008 | NR | relapse prevention theory | yes | yes | no |
| Bold | 2016 | The cognitive-behavioural relapse model (Marlatt & Gordon, 1985) | negative reinforcement model | yes | yes | no |
| Bold | 2016 | The negative reinforcement model of drug addiction (Baker et al., 2004) | relapse prevention theory | yes | yes | no |
| Bolman | 2018 | The relapse prevention theory (Marlatt & Donovan, 2005) | relapse prevention theory | yes | yes | no |
| Brodbeck | 2013 | The cognitive-behavioural relapse model by Marlatt and Gordon (1985) | relapse prevention theory | yes | yes | no |
| Brodbeck | 2014 | The cognitive-behavioural relapse model by Marlatt and Gordon (1985) |  |  |  |  |
| Businelle | 2016 | NR | relapse prevention theory | yes | yes | no |
| Cambron | 2019 | Witkiewitz and Marlatt’s (2004) dynamic model of relapse prevention |  |  |  |  |
| Cambron | 2020 | NR | model of absentminded lapses | yes | yes | no |
| Catley | 2000 | The model of absentminded lapses (Tiffany, 1990) |  |  |  |  |
| Cofta-Woerpel | 2011 | NR |  |  |  |  |
| Dermody | 2020 | NR | expectancy violation theory | yes | yes | no |
| Derrick | 2018 | Expectancy Violation Theory (Burgoon, 1995; Burgoon & Hale, 1988) | attentional bias theory | yes | yes | no |
| Etcheverry | 2016 | Attentional bias |  |  |  |  |
| Ferguson | 2006 | NR | model of absentminded lapses | yes | yes | no |
| Ferguson & Shiffman | 2010 | The model of absentminded lapses (Tiffany, 1990) | social learning theory | yes | yes | yes |
| Gwaltney | 2005a | Social-Learning Relapse Models (Shiffman, 1989) | social learning theory | yes | yes | no |
| Gwaltney | 2005b | Social-Learning Relapse Models (Shiffman, 1989) | relapse prevention theory | yes | yes | no |
| Gwaltney | 2002 | Relapse models (Marlatt & Gordon, 1985) |  |  |  |  |
| Kirchner | 2013 | NR |  |  |  |  |
| Koslovsky | 2018 | NR |  |  |  |  |
| Lam | 2014 | NR |  |  |  |  |
| Langdon | 2016 | NR | strength model of self-regulation | yes | yes | no |
| Liu | 2013 | The strength model of self-regulation (Piasecki et al., 2002) |  |  |  |  |
| McCarthy | 2008 | NR |  |  |  |  |
| McCarthy | 2010 | NR |  |  |  |  |
| McCarthy | 2006 | NR |  |  |  |  |
| Messer | 2018 | NR |  |  |  |  |
| Minami | 2017 | NR | negative reinforcement model | yes | yes | no |
| Minami | 2010 | Negative reinforcement models | relapse prevention theory | yes | yes | no |
| Minami | 2015 | High-risk contexts (Shiffman et al., 1996) | negative reinforcement model; broaden-and-build theory of positive emotions |  |  |  |
| Minami | 2014 | The modified negative reinforcement drug motivation model (Baker et al., 2004), the broaden-and-build model (Fredrickson, 2000; 2003), and the positive affect model (Wagner & Ingersoll, 2008) | reversal theory | yes | yes | no |
| O'Connell | 2000 | Reversal theory (Smith & Apter, 1975) | relapse prevention theory | yes | yes | no |
| O'Connell | 2002 | Anticipatory coping (e.g., stimulus control, symptom prevention, social support) | relapse prevention theory | yes | yes | no |
| O'Connell | 2007 | Coping with temptations | strength model of self-regulation | yes | yes | no |
| O'Connell | 2008 | Resource depletion model of self-control (Muraven & Baumeister, 2000) | strength model of self-regulation | yes | yes | no |
| O'Connell | 2008 | Resource depletion model of self-control (Muraven & Baumeister, 2000) | classic conditioning | yes | yes | no |
| O'Connell | 2010 | Classical (Pavlovian) conditioning | classic conditioning | yes | yes | no |
| O'Connell | 2010 | Classical (Pavlovian) conditioning | reversal theory | yes | yes | no |
| O’Connell | 2004 | Reversal theory (Smith & Apter, 1975) |  |  |  |  |
| Peters | 2009 | NR |  |  |  |  |
| Shiffman | 2007 | NR | negative reinforcement model | yes | yes | no |
| Shiffman | 2008 | Classic theories of addiction, which propose that people smoke for relief from conditioned and unconditioned NA states (Solomon & Corbit, 1974; Wikler, 1948) | episodic model of relapse | yes | yes | no |
| Shiffman | 1996 | The episodic model of relapse (Shiffman, 1989) |  |  |  |  |
| Spears | 2019 | NR |  |  |  |  |
| Suchting | 2019 | NR |  |  |  |  |
| Vasilenko | 2014 | NR | broaden-and-build theory of positive emotions | yes | yes | no |
| Vinci | 2017 | The broaden-and-build model (Fredrickson, 2000; 2003) and the dynamic model of affect (Zautra et al., 2001) |  |  |  |  |
| Waring | 2020 | NR | negative reinforcement model | yes | yes | no |
| Yeh | 2012 | The negative reinforcement model of drug motivation (Baker et al., 2004) |  |  |  |  |
| Buitenhuis | 2021 | NR | relapse prevention theory | yes | yes | no |
| Cambron | 2020 | Social cognitive models (e.g., Witkiewitz & Marlatt, 2004) |  |  |  |  |
| Hebert | 2021 | NR |  |  |  |  |
| Nakajima | 2020 | NR | relapse prevention theory | yes | yes | no |
| Potter | 2021 | Marlatt and Gordon's (1985) relapse prevention model | relapse prevention theory | yes | yes | no |
| Shiffman | 2020 | NR | relapse prevention theory | yes | yes | no |
| Shiffman | 2020 | Marlatt and Gordon's (1985) relapse prevention model |  |  |  |  |
| Chakraborti | 2022 | Marlatt’s Cognitive-Behavioral Theory | relapse prevention theory | yes | yes | no |
| Liang | 2021 | NR |  |  |  |  |
| Schultz | 2021 | Negative reinforcement models | negative reinforcement model | yes | yes | yes |
| Vinci | 2021 | Brondolo’s biopsychosocial model of perceived discrimination | biopsychosocial model of perceived discrimination | yes | yes | no |
| Majmundar | 2020 | NR |  |  |  |  |
| Shiffman | 1997 | Nicotine withdrawal and conditioned compensatory responses | negative reinforcement model | yes | yes | no |

*Note.* NR = Not reported.

***Moderator analyses***

*Environmental and social cues*

| **Moderators** | **OR (95% CI)** | **p-value** |
| --- | --- | --- |
| Intercept | 0.23 (0-1252664.13) | 0.85 |
| Mean age | 0.99 (0.73-1.34) | 0.94 |
| Female sex percentage | 1.01 (0.94-1.09) | 0.76 |
| Ethnicity white percentage | 1.00 (0.97-1.03) | 0.90 |
| Mean CPD | 1.21 (0.91-1.61) | 0.19 |
| EMA study type - Interventional | 0.31 (0.04-2.67) | 0.28 |
| Study duration (days) | 0.98 (0.88-1.09) | 0.68 |
| Random slope within | 0.52 (0.04-6.64) | 0.62 |

*Cravings*

| **Moderators** | **OR (95% CI)** | **p-value** |
| --- | --- | --- |
| Intercept | 39172449945.7 (14211.69-107973108364167344) | <0.01 |
| Mean age | 0.60 (0.43-0.83) | <0.01 |
| Female sex percentage | 0.97 (0.93-1.01) | 0.20 |
| Ethnicity white percentage | 1.03 (1.01-1.05) | <0.001 |
| Mean CPD | 0.91 (0.84-1) | 0.05 |
| EMA study type - Interventional | 1.84 (0.96-3.56) | 0.07 |
| Study duration (days) | 0.98 (0.94-1.02) | 0.23 |
| Incentive schedule – Flat payment | 0.00 (0.00-0.04) | <0.01 |
